# Impact of Adiposity on Cardiopulmonary Fitness in Children and Adolescents with Ventricular Septal Defects: a CPET-Based Comparative Study

**DOI:** 10.7150/ijms.121548

**Published:** 2025-09-29

**Authors:** I-Hsiu Liou, Guan-Bo Chen, Shu-Fen Sun, Ruei-Sian Ding, Wan-Yun Huang, Sheng-Hui Tuan

**Affiliations:** 1 Department of Physical Therapy, Fooyin University, Kaohsiung, Taiwan (R.O.C.).; 2 Department of Physical Medicine and Rehabilitation, Kaohsiung Veterans General Hospital, Kaohsiung, Taiwan (R.O.C.).; 3 Department of Internal Medicine, Kaohsiung Armed Forces General Hospital, Kaohsiung, Taiwan (R.O.C.).; 4 School of Medicine, National Yang Ming Chiao Tung University, Taipei, Taiwan (R.O.C.).; 5 Department of Rehabilitation Medicine, Cishan Hospital, Ministry of Health and Welfare, Kaohsiung, Taiwan (R.O.C).; 6 Department of Physical Medicine and Rehabilitation, School of Medicine, College of Medicine, Kaohsiung Medical University, Kaohsiung, Taiwan (R.O.C.).

**Keywords:** body mass index, cardiopulmonary exercise testing, cardiopulmonary fitness, obesity, peak oxygen consumption, ventricular septal defect

## Abstract

**Background:** Ventricular septal defect (VSD) is the most common congenital heart defect in children. While previously considered benign, recent studies suggest long-term impacts on cardiopulmonary fitness (CPF). Overweight and obesity, increasingly prevalent among children, may further impair CPF in this population. This study aimed to evaluate the relationship between adiposity and CPF in children with VSD using cardiopulmonary exercise testing (CPET).

**Methods:** This retrospective study included 349 children and adolescents with VSD and 349 age-, sex-, and body mass index (BMI)-matched healthy controls. Participants underwent symptom-limited treadmill CPET. Children with VSD were stratified into BMI categories (underweight, normal, overweight, obesity) based on national standards. Multiple CPET parameters were analyzed, including anaerobic threshold metabolic equivalents (AT MET) and peak metabolic equivalents (peak MET).

**Results:** Children with VSD had significantly higher rates of both underweight (15.5% vs. 4.3%) and obesity (14.6% vs. 9.7%) compared to controls (p < 0.001). Within the VSD group, AT MET and peak MET declined progressively with increasing BMI. [AT MET: 7.41 ± 1.57 (underweight), 6.86 ± 1.38 (normal), 6.01 ± 1.23 (overweight), 5.62 ± 1.23 (obese), p < 0.001; Peak MET: 10.37 ± 2.22 (underweight), 9.58 ± 1.94 (normal), 8.56 ± 1.70 (overweight), 7.81 ± 1.60 (obese), p < 0.001]. Compared to controls, children with VSD showed lower AT MET (6.56 ± 1.51 vs. 6.97 ± 1.47, p < 0.001) and peak MET (9.32 ± 2.03 vs. 10.17 ± 1.95, p < 0.001), along with reduced peak heart rate and heart rate at AT.

**Conclusion:** Children with VSD, regardless of surgical status, exhibit diminished CPF compared to healthy peers. Moreover, both undernutrition and excessive adiposity are more prevalent in the VSD group. Obesity was associated with significantly impaired cardiopulmonary fitness, highlighting the need for early identification and lifestyle interventions. Routine CPET and weight management strategies should be incorporated into long-term care for pediatric VSD patients.

## 1. Introduction

Ventricular septal defect (VSD) is the most prevalent congenital heart disease (CHD) in children, accounting for approximately 40% of all CHD cases^1^. The global birth prevalence has been reported to reach up to 2.62 per 1,000 live births^2^, with a corresponding prevalence of approximately 4.01 per 1,000 live births in Taiwan^3^. VSDs were previously considered hemodynamically insignificant^4^. Historically, VSDs, whether surgically repaired during childhood or left unrepaired due to hemodynamic insignificance, were considered benign conditions with minimal long-term impact on adult health. However, accumulating evidence over the past decade has revealed a spectrum of adverse outcomes in adults with VSD, including reduced functional capacity^5^, ^6^, impaired pulmonary function^7^, diminished exercise-induced cardiac output^8^, and altered heart rate variability^9^. As a result, the 2018 clinical practice guidelines issued by the American College of Cardiology (ACC) and the American Heart Association (AHA) were updated to recommend lifelong follow-up for individuals with VSD.^10^. One recent study in Taiwan has shown that children with VSD, regardless of surgical status, exhibit lower peak oxygen consumption (peak VO₂), minute ventilation, and anaerobic threshold (AT) when compared to age- and sex-matched healthy peers^11^. This suggests subtle but persistent deficits in cardiopulmonary fitness (CPF).

Emerging evidence indicates that children with surgically closed VSDs may exhibit poorer ventilatory responses and blunted chronotropic adaptation during exercise compared to those with unrepaired lesions^6^, ^11^. This may stem from altered pulmonary mechanics or surgical impact on autonomic pathways, potentially limiting cardiac output and reducing exercise capacity compared to those with unrepaired VSDs. These findings underscore the complexity of distinguishing between the long-term cardiopulmonary outcomes of repaired and unrepaired VSDs and highlight the need for ongoing surveillance.

Childhood obesity has become a major public health concern both globally and locally, with its prevalence steadily increasing over recent decades. It arises from an imbalance between energy intake and expenditure and is influenced by a complex interplay of genetic, behavioral, and environmental factors. Childhood obesity is associated with a wide range of adverse physical, psychological, and social health outcomes^12^. Children with CHD are increasingly susceptible to developing obesity, particularly as they age and are exposed to socioeconomic or behavioral risk factors that compound their cardiometabolic burden^13^. Although children with moderate to complex CHD initially have lower odds of obesity, their predicted probability of becoming developing obesity approaches that of the general population with increasing age^13^. In this population, physical activity restrictions, altered metabolic demands, and suboptimal feeding practices may further elevate the risk of excessive weight gain and its associated cardiovascular consequences^14^. Reported prevalence of obesity in children with CHD ranges from 16% to over 25%, with an upward trend over time^14^. A growing body of literature highlights the impact of obesity on CPF in the pediatric population. Overweight and obesity are associated with lower peak VO₂, shorter exercise time, and higher ventilatory equivalent for CO₂ (VE/VCO₂ slope) in both healthy^15^-^17^ and CHD populations^18^-^20^. Given these findings, it is plausible that body fat may further impair CPF in children with VSD. However, the literature remains sparse, especially concerning objective assessments such as cardiopulmonary exercise testing (CPET). Therefore, the aims of this study were to: (1) compare the prevalence of obesity between children and adolescents with VSD and healthy controls; (2) examine differences in body composition between those with repaired and unrepaired VSD; and (3) investigate the association between body mass index (BMI) categories and cardiopulmonary fitness using CPET-derived metrics.

## 2. Materials and Methods

### 2.1. Study design and participants

This retrospective cohort study was conducted at a single tertiary medical center in Kaohsiung City, southern Taiwan. The study population consisted of children aged 6 to 18 years who attended the pediatric cardiology outpatient clinic for VSD follow-up between July 2019 and June 2024. Both patients with surgically repaired VSD and those with unrepaired lesions deemed hemodynamically insignificant based on prior echocardiographic or catheterization assessments were eligible for inclusion. Additional enrollment criteria required completion of a comprehensive transthoracic echocardiogram, a standard 12-lead electrocardiogram, body composition assessment, and a symptom-limited treadmill cardiopulmonary exercise test. Children were excluded if they had other structural heart anomalies (e.g., atrial septal defect or patent ductus arteriosus), moderate to severe valvular dysfunction, clinically significant arrhythmias, ventricular hypertrophy, or any respiratory or systemic condition potentially affecting cardiopulmonary function.

A control group was assembled by matching healthy peers by age, sex, and BMI. These children had been referred to the same clinic during the same time frame for complaints such as exertional dyspnea or chest discomfort, and all had undergone cardiopulmonary exercise testing without pathological findings. All control participants underwent transthoracic echocardiography, which confirmed the absence of congenital or structural heart disease and revealed no pathological findings.

Data collected included age, sex, height, weight, and percentage of body fat. The study complied with the ethical standards of the Declaration of Helsinki and was approved by the Institutional Review Board of Kaohsiung Veterans General Hospital (IRB number: VGHKS17-CT11-11). The research also followed the STROBE (Strengthening the Reporting of Observational Studies in Epidemiology) reporting guidelines. As this was a retrospective study, no formal sample size calculation was performed. To maximize statistical power and reduce selection bias, we included all eligible children with complete clinical and CPET data collected between July 2019 and June 2024.

### 2.2. Anthropometry-body composition

Standing height was obtained using a calibrated wall-mounted stadiometer (DETECTO, Model 3PHTROD-WM, Webb City, MO, USA), with participants barefoot and maintaining upright alignment. Body composition parameters—including body fat percentage, total body water, appendicular skeletal muscle mass, and bone mass—were evaluated using the Zeus 9.9 PLUS system (Jawon Medical Co., Ltd., Gyeongsangbuk-do, Republic of Korea) via multifrequency bioelectrical impedance analysis using a tetrapolar electrode configuration. BMI was automatically derived by dividing body weight (kg) by height squared (m²). Based on BMI values, participants were categorized into normal or excessive adiposity groups, using age- and sex-specific percentile thresholds established by Taiwan's Ministry of Health and Welfare, where overweight and obesity are defined as BMI above the 85th and 95th percentiles, respectively^21^.

### 2.3 Cardiopulmonary exercise testing

Participants completed a symptom-limited CPET on a treadmill using a metabolic system equipped with a gas analyzer, airflow module, and ECG monitoring (Metamax 3B; Cortex Biophysik GmbH, Leipzig, Germany). Measurements were captured every 30 seconds to monitor dynamic physiological responses. Before testing, a licensed physical therapist provided a standardized orientation to familiarize each subject with the protocol and equipment. The CPET followed the Bruce ramp protocol, in accordance with guidelines set by the American College of Sports Medicine (ACSM)^22^. We terminated the CPET when participants exhibited maximal exertion, defined as achieving one or more criteria established by the ACSM, including a respiratory exchange ratio (RER) ≥ 1.1, a plateau in oxygen consumption, or attainment of ≥ 85% of age-predicted maximal heart rate. Testing was also discontinued if subjects reported intolerable symptoms or voluntary exhaustion. An experienced physiatrist (I.-H.L.) with over a decade of expertise in CPET supervised the sessions. Breath-by-breath analysis was used to determine oxygen uptake (VO_2_) and carbon dioxide output (VCO₂), while heart rate, blood pressure, and RER were monitored throughout. The peak rate pressure product (PRPP), an index of cardiac workload, was calculated as the product of peak systolic blood pressure and peak heart rate^23^. Anaerobic threshold (AT) was identified using ventilatory equivalents for oxygen (VE/VO₂) and carbon dioxide (VE/VCO₂)^24^. The physiatrist also determined the oxygen consumption at AT (AT VO₂) and at peak exercise (peak VO₂). Metabolic equivalents (METs) were calculated by dividing VO₂ by 3.5 mL/kg/min, and oxygen pulse was defined as peak VO₂ divided by peak heart rate. Heart rate recovery (HRR) is measured as the difference between the peak heart rate achieved during exercise and the heart rate at 1 minute post-exercise. HRR is a well-established, non-invasive marker of autonomic nervous system function and cardiovascular health^25^.

### 2.4. Statistical analysis

All statistical analyses were performed using IBM SPSS Statistics for Windows, version 25.0 (IBM Corp., Armonk, NY, USA; released 2017). Continuous variables are presented as mean ± standard deviation (SD), and categorical variables are expressed as frequency and percentage. Prior to inferential analyses, the assumptions of normality and homogeneity of variance were evaluated using the Shapiro-Wilk test and Levene's test, respectively.

For group comparisons, different statistical tests were applied based on data distribution characteristics. Specifically, independent t-tests were used for normally distributed continuous variables, while the Mann-Whitney U test was applied to non-normally distributed variables. Chi-square tests were used to compare proportions between groups (e.g., BMI categories, sex distribution, and adiposity classifications). For analyses involving more than two groups, such as comparisons among different BMI categories within the VSD cohort, one-way analysis of variance (ANOVA) was used to evaluate overall group differences for normally distributed variables. Post-Hoc analysis was done by Bonferroni correction to adjust for multiple testing and identify specific group pairs with statistically significant differences. The Bonferroni method adjusts the alpha level by dividing 0.05 by the number of pairwise comparisons. A two-tailed p-value < 0.05 was considered statistically significant for all analyses.

## 3. Results

### 3.1. Study population and baseline characteristic comparison

A total of 366 children and adolescents with a history of VSD met the inclusion criteria. Of these, 17 were excluded: one with moderate and one with severe valvular heart disease, two with significant arrhythmias, seven with missing or incomplete body composition data, and six with incomplete CPET results. Ultimately, 349 children and adolescents with VSD, along with 349 age-, sex-, and BMI-matched healthy controls, were included for baseline comparisons. The mean age of children and adolescents with VSD was 12.51 ± 3.46 years, with boys accounting for 46.99% of the cohort.

As shown in Table 1, there were no significant differences between the VSD and control groups in terms of age, height, weight, systolic and diastolic blood pressure, or resting heart rate. However, children with VSD exhibited significantly higher BMI (20.14 ± 4.61 vs. 19.26 ± 3.11 kg/m², p < 0.007) and body fat percentage (19.31 ± 8.14% vs. 17.15 ± 6.29%, p < 0.001) than controls. In addition, the overall distribution of BMI categories differed significantly between groups (p < 0.001), with the VSD group showing a higher proportion of individuals with underweight (15.5% vs. 4.3%, p < 0.001) and a greater proportion of participants classified as having obesity (14.6% vs. 9.7%). When comparing the repaired (n = 217) and unrepaired (n = 132) VSD subgroups, no significant differences were found in age, height, BMI, or body fat percentage (p > 0.05 for all). Although children with unrepaired VSD tended to be taller and heavier than those with repaired defects, these differences did not reach statistical significance (height: p = 0.075; weight: p = 0.053). The distribution of BMI categories was also comparable between the two subgroups.

### 3.2 Comparison of cardiopulmonary exercise performance between children and adolescents with ventricular septal defect and controls, and between repaired and unrepaired VSD subgroups

CPET was conducted on 349 children and adolescents with VSD and 349 controls. As summarized in Table 2, children and adolescents with VSD had significantly lower heart rate at AT (AT HR, 139.38 ± 15.7 vs. 143.4 ± 12.21 bpm, p < 0.001), AT MET (6.56 ± 1.51 vs. 6.97 ± 1.47, p < 0.001), peak heart rate (peak HR, 174.72 ± 16.14 vs. 178.67 ± 16.29 bpm, p = 0.001), peak MET (9.32 ± 2.03 vs. 10.17 ± 1.95, p < 0.001), and peak RER (1.17 ± 0.10 vs. 1.19 ± 0.11, p = 0.002) compared to the control group. No significant differences were found in peak VO₂, VE, PRPP, or HRR.

Further subgroup analysis comparing children with repaired and unrepaired VSDs revealed that the unrepaired group had significantly higher peak HR than the repaired group (177.49 ± 9.91 vs. 172.98 ± 18.86 bpm, p = 0.004), and higher peak RER (1.18 ± 0.11 vs. 1.16 ± 0.09, p = 0.019). Interestingly, HRR was significantly higher in the unrepaired group compared to the repaired group (28.96 ± 11.39 vs. 25.86 ± 10.79, p = 0.033). No other CPET parameters showed significant differences between these two subgroups.

### 3.3 Cardiopulmonary exercise performance in children and adolescents with VSD stratified by body mass index categories

To evaluate the impact of body composition on CPF, we stratified participants with VSD into four BMI categories: participants with underweight, normal weight, overweight, and obesity. As shown in Table 3, significant between-group differences were observed across several CPET parameters. AT MET progressively declined as BMI increased, with the highest value observed in the group of participants with underweight (7.41 ± 1.57) and the lowest in group of participants with obesity (5.62 ± 1.23), reaching statistical significance across all BMI categories (p < 0.001). Post hoc analysis revealed that AT MET was significantly lower in the group of participants with normal, overweight, and obese compared to the of participants with underweight, and significantly lower in both the group of participants with overweight and obese compared to those in the group of normal BMI.

A similar trend was noted for peak MET, which decreased from 10.37 ± 2.22 in the group of participants with underweight to 7.81 ± 1.60 in the group of participants with obesity (p < 0.001). Pairwise comparisons demonstrated that the groups of participants with overweight and obese had significantly lower peak MET values than both the group of participants with underweight and normal BMI. Interestingly, AT VO₂ and peak VO₂ were significantly higher in the group of participants with obesity compared to both the group of participants with underweight and normal BMI (p < 0.001 for AT VO_2_, p = 0.002 for peak VO₂). This finding likely reflects absolute oxygen uptake influenced by greater body mass, rather than superior aerobic fitness. HRR differed significantly across BMI categories (p = 0.012), with post hoc comparisons indicating a significantly lower HRR in the group of participants with overweight compared to the those with normal BMI. No significant differences were found among groups in peak HR, peak RER, or PRPP.

## 4. Discussion

This single-center retrospective study evaluated CPF in children and adolescents with VSD using CPET. Several key findings emerged from our analysis. First, despite advancements in medical and surgical management, children and adolescents with VSD, regardless of repair status, exhibited impaired CPF, as evidenced by significantly lower AT MET and peak MET values compared to healthy controls. Second, the cohort with VSD demonstrated disproportionately higher rates of both underweight and obesity relative to their healthy peers. Third, excessive adiposity, defined by overweight and obesity based on BMI classification, was associated with significantly poorer CPET performance, indicating an additional burden on exercise capacity in this population.

### 4.1. Impaired cardiopulmonary fitness in children and adolescents with VSD

Our findings showed that children and adolescents with VSD had significantly lower AT MET and peak MET than healthy controls, consistent with prior studies reporting reduced exercise capacity in this population. Although early research yielded conflicting results—such as those by Meijboom et al. who found 84% of children with VSD to have normal exercise capacity based on treadmill testing^26^, and Binkhorst et al. who reported similar peak VO₂ levels between patients with VSD and controls^27^, our findings align more closely with more recent and comprehensive literature. Reybrouck et al. demonstrated that children exhibited reduced exercise performance shortly after VSD repair^28^, and Nederend et al. further demonstrated sustained reductions in exercise capacity even 10 years after surgical closure^29^. Furthermore, Yenny et al. conducted a systematic review and meta-analysis that confirmed significantly lower peak VO₂ and exercise tolerance in children with simple CHD, including those with VSD, compared to healthy peers^19^. Our study differs from earlier work by including a large, diverse cohort of children and adolescents with both surgically repaired and unrepaired defects, thereby offering a broader picture of CPF across the VSD spectrum.

The mechanisms underlying reduced exercise capacity in patients with VSD remain multifactorial. Several studies suggest that elevated pulmonary vascular resistance during exercise may lead to increased right ventricular (RV) afterload. For example, enhanced right ventricular systolic pressure and retrograde pulmonary artery flow under incremental workloads have been reported in patients with VSD^8^, ^30^. Additionally, enlarged RV end-diastolic volume, observed through imaging, implies impaired RV compliance and increased filling pressures. Exercise echocardiography has further revealed abnormal ventricular contractile responses in both patients with repaired and unrepaired VSD, potentially contributing to their diminished CPF^31^, ^32^. These hemodynamic alterations, such as higher pulmonary vascular resistance, abnormal RV loading, and impaired contractility, may collectively explain the chronotropic and ventilatory inefficiencies observed during CPET in this population.

### 4.2 Increased prevalence of underweight and obesity in patients with VSD

A striking finding in our study is the dual burden of malnutrition in the cohort with VSD, characterized by increased rates of both underweight and obesity compared to matched controls. It was not surprising to observe that children and adolescents with VSD had higher prevalence of having obesity. Recent evidence from a systematic review demonstrated that the prevalence of overweight in children with CHD ranged from 9.5% to 31.5%, and obesity from 9.5% to 26.2%^33^. While many studies reported comparable overweight/obesity rates between CHD and control groups^34^, ^35^, others have shown higher obesity prevalence in populations with CHD, particularly those with non-cyanotic lesions such as VSD^14^, ^33^. Children with CHD also had greater waist circumference than controls after adjusting for sex, birth weight, physical activity, and lean mass. Interestingly, overweight and obesity were more prevalent in boys with CHD than girls with CHD^33^.

Contrary to expectations, children and adolescents with VSD also had higher prevalence of being underweight. Our findings corroborate previous reports that Asian cohorts with CHD may initially present with higher underweight prevalence in childhood, shifting toward increased overweight/obesity in adolescence. One large Taiwanese study showed that younger children with CHD had lower overweight/obesity rates and higher underweight prevalence compared to peers, especially in those with cyanotic CHD. However, approximately one-third of adolescent boys with CHD and one-fifth of girls with CHD were either overweight or obese, highlighting a significant long-term health burden^36^. This U-shaped distribution reflects a complex interplay between early growth restriction, metabolic demands, surgical correction, and lifestyle factors^14^, ^33^. Given the potential cardiovascular consequences of both undernutrition and excess adiposity, early identification and targeted nutritional and physical activity interventions should be prioritized in this vulnerable population.

### 4.3. Influence of BMI on cardiopulmonary fitness in children and adolescents with VSD

Stratified analyses revealed a clear inverse relationship between BMI and exercise capacity among children and adolescents with VSD. Both AT MET and peak MET decreased significantly from the underweight to the obese categories. These findings are consistent with prior studies demonstrating that overweight and obesity are associated with significantly shorter exercise time and lower performance, regardless of CHD severity^18^.

The pathophysiology behind this association is multifaceted. Obesity has been shown to impair endothelial function and increase systemic inflammation, even in children^37^. These metabolic and vascular abnormalities are of particular concern in individuals with CHD, who may already have compromised cardiovascular function. Moreover, elevated baseline blood pressure in overweight and obese children with CHD has been reported, further contributing to cardiovascular strain^18^, ^38^. In addition, the observed reduction in HRR among overweight children and adolescence with VSD may signal impaired autonomic recovery, which has been linked to elevated cardiovascular risk in pediatric and adult populations^25^. Physical activity restrictions, whether self-imposed, parentally reinforced, or medically advised, frequently limit energy expenditure in this population. Studies have shown that many children with CHD abstain from physical activities unnecessarily, partly due to overprotective parenting and inconsistent exercise guidance from healthcare professionals^39^, ^40^. Together, these findings suggest that the influence of BMI on CPF in children and adolescents with VSD is shaped by both physiological limitations and modifiable behavioral factors. A multidisciplinary approach that incorporates cardiology, nutrition, physical therapy, and psychological support may be essential to optimizing outcomes in this population.

### 4.4 Repaired vs. Unrepaired VSD

In our study, we found that both patients with repaired and unrepaired VSD exhibited impaired CPF, with no significant differences in key CPET parameters such as peak VO₂ and AT MET between the two groups. Studies reported that children with both repaired and small unrepaired VSDs show reduced exercise tolerance compared to healthy controls, and this impairment may persist or even worsen with age^5^, ^28^, ^29^. This finding suggests that even small, hemodynamically insignificant unrepaired VSDs may contribute to exercise limitations in pediatric populations. Importantly, we observed significantly lower AT MET among patients with VSD, irrespective of repair status. Since AT reflects submaximal oxygen uptake and is less affected by voluntary effort than peak MET^41^, this finding provides robust evidence of early exercise intolerance and cardiovascular insufficiency in meeting tissue oxygen demands during exertion.

Interestingly, despite differences in clinical management, we observed no significant difference in BMI distribution between children and adolescents with repaired and unrepaired VSD, suggesting that nutritional status and body composition are more strongly influenced by common environmental and behavioral factors (e.g., feeding practices, physical activity limitations) than by surgical intervention alone. This underscores the need for uniform cardiopulmonary monitoring and lifestyle counseling across all patients with VSD, regardless of repair status.

### 4.5 Study limitations

Several limitations of this study should be acknowledged. First, this was a single-center retrospective analysis conducted in southern Taiwan, which may limit the generalizability of our findings to broader or ethnically diverse populations. Second, the retrospective design limited our ability to control for behavioral and lifestyle confounders, such as dietary habits, physical activity levels, and socioeconomic status that may have influenced both adiposity and cardiopulmonary fitness. Specifically, we lacked data on participants' activity levels, self-rated fitness, dietary behaviors, and socioeconomic background, precluding a more comprehensive analysis of these potential influences. Future prospective studies should incorporate objective measures of physical activity and sedentary behavior, given their potential impact on both adiposity and exercise capacity in children with VSD. Third, echocardiographic parameters such as pulmonary-to-systemic blood flow ratio or pulmonary artery pressures were not available, limiting our ability to explore structural or hemodynamic correlates of exercise impairment. Lastly, while our cohort is one of the largest to date examining VSD and cardiopulmonary exercise performance, prospective, multi-center studies with standardized physiological and lifestyle measurements are warranted to confirm and extend these findings.

## 5. Conclusion

Our study highlights the substantial negative impact of excess adiposity on CPF in children and adolescents with VSD. Obesity was associated with significantly reduced anaerobic threshold and peak exercise capacity, underscoring the importance of early identification and intervention to optimize body composition in this vulnerable population. In addition, children and adolescents with VSD, regardless of surgical repair status, demonstrated poorer CPF compared to age-matched healthy controls. These findings support the incorporation of routine CPF assessments and individualized lifestyle strategies into the longitudinal care of pediatric patients with VSD, and call for further studies to explore the mechanisms linking obesity and exercise intolerance in children and adolescents with VSD.

## Figures and Tables

**Table 1:** Baseline characteristics of children and adolescents with a history of ventricular septal defect compared to controls, and between repaired and unrepaired VSD subgroups.

		VSD n=349	Control n=349	*p* value	Repaired VSD n=217	Unrepaired VSD n=132	*p* value
Age (years)		12.51 ± 3.46	12.54 ± 3.45	0.913	12.44 ± 3.57	12.62 ± 3.27	0.635
**Sex**	M	164 (46.99%)	164 (46.99%)	1.000	99 (45.62%)	61 (46.21%)	0.998
	F	185 (53.01%)	185 (53.01%)		118 (54.38%)	71 (53.79%)	
Height (cm)		149.91 ± 17.6	151.37 ± 18.25	0.283	148.59 ± 17.3	152.02 ± 17.93	0.075
Weight (kg)		46.84 ± 17.36	45.43 ± 15.96	0.311	45.42 ± 16.41	49.09 ± 18.61	0.053
BMI (kg/m^2^)		20.14 ± 4.61	19.26 ± 3.11	<0.007*	19.86 ± 4.42	20.59 ± 4.88	0.148
Body fat (%)		19.31 ± 8.14	17.15 ± 6.29	<0.001*	18.93 ± 8.37	19.92 ± 7.76	0.270
U(%)		54 (15.5%)	15 (4.3%)	<0.001^*,a^	38 (17.5%)	17 (12.9%)	0.401^a^
N (%)		193 (55.3%)	248 (71.1%)		117 (53.9%)	76 (57.5%)	
O (%)		51 (14.6%)	52 (14.9%)		35 (16.1%)	17 (12.9%)	
F (%)		51 (14.6%)	34 (9.7%)		27 (12.5%)	22 (16.7%)	
Rest SBP		112.66 ± 17.31	111.18 ± 16.12	0.241	111.71 ± 17.72	114.18 ± 16.59	0.192
Rest DBP		66.63 ± 9.87	66.41 ± 8.95	0.760	66.36 ± 9.92	67.04 ± 9.81	0.529
Rest HR		85.08 ± 14.83	86.11 ± 13.33	0.335	84.96 ± 15.17	85.29 ± 14.32	0.840

M: male; F: female; BMI: body mass index; U (%): percentage of underweight subjects; N (%): percentage of normal weight subjects; O (%): percentage of overweight subjects; F (%): percentage of obesity subjects; SBP: systolic blood pressure; DBP: diastolic blood pressure; HR: heart rate ^a^ All the comparisons between girls and boys were done by independent t test except p values that marked with ^a^, which was analyzed by independent Chi square test for comparison percentage of excessive adiposity between children with VSD and control p value < 0.05

**Table 2:** Comparison of cardiopulmonary exercise performance between children and adolescents with ventricular septal defect and controls, and between repaired and unrepaired VSD subgroups.

	VSD n=349	Control n=349	*p* value	Repaired VSD n=217	Unrepaired VSD n=132	*p* value
AT HR (bpm)	139.38 ± 15.7 (137.73-141.03)	143.4 ± 12.21 (142.11-144.69)	<0.001*	138.97 ± 15.25 (136.93-141.01)	140.04 ± 16.42 (137.21-142.87)	0.533
AT MET	6.56 ± 1.51 (6.40-6.72)	6.97 ± 1.47 (6.82-7.12)	<0.001*	6.66 ± 1.59 (6.45-6.87)	6.38 ± 1.36 (6.15-6.61)	0.091
ATVO_2_ (ml/kg/min)	1036.90 ± 366.80 (998.28-1075.52)	1015.44 ± 339.24 (979.72-1051.16)	0.422	1022.15 ± 371.7 (972.42-1071.88)	1060.28 ± 359.02 (998.46-1122.10)	0.345
Peak HR (bpm)	174.72 ± 16.14 (173.02-176.42)	178.67 ± 16.29 (176.95-180.39)	0.001*	172.98 ± 18.86 (170.46-175.50)	177.49 ± 9.91 (175.78-179.20)	0.004*
Peak MET	9.32 ± 2.03 (9.11-9.53)	10.17 ± 1.95 (9.96-10.38)	<0.001*	9.39 ± 2.19 (9.10-9.68)	9.21 ± 1.76 (8.91-9.51)	0.416
PeakVO_2_ (ml/kg/min)	1484.10 ± 543.71 (1426.86-1541.34)	1492.88 ± 516.40 (1438.51-1547.25)	0.826	1449.87 ± 541.32 (1377.44-1522.30)	1538.46 ± 545.06 (1444.61-1632.31)	0.137
Peak VE	41.77 ± 13.26 (40.37-43.17)	43.01 ± 13.61 (41.58-44.44)	0.223	41.72 ± 13.63 (39.90-43.54)	41.87 ± 12.7 (39.68-44.06)	0.919
Peak RER	1.17 ± 0.10 (1.16-1.18)	1.19 ± 0.11 (1.18-1.20)	0.002*	1.16 ± 0.09 (1.15-1.17)	1.18 ± 0.11 (1.17-1.20)	0.019*
PRPP	28598.34 ± 5954.67 (27971.43-29225.25)	28523.31 ± 6256.91 (27864.58-29182.04)	0.871	28361.35 ± 6245.91 (27525.64-29197.06)	28969.51 ± 5469.31 (28027.78-29911.24)	0.353
HRR	27.04 ± 11.1 (25.87-28.21)	26.33 ± 9.43 (25.34-27.32)	0.423	25.86 ± 10.79 (24.42-27.30)	28.96 ± 11.39 (27.48-30.92)	0.033*

AT HR: heart rate at anaerobic threshold; AT MET: metabolic equivalent at anaerobic threshold; Peak HR: peak hear rate during exercise testing; Peak MET: peak metabolic equivalent during exercise testing; AT VO_2_: oxygen consumption at anaerobic threshold; Peak VO_2_: peak oxygen consumption during exercise testing; VE: minute ventilation; RER: respiratory exchange ratio; PRPP: peak heart rate pressure product; HRR: heart rate reserve *p value < 0.05

**Table 3:** Cardiopulmonary exercise performance in children and adolescents with VSD stratified by body mass index categories.

	Underweight n=54	Normal n=193	Overweight n=51	Obesity n=51	*p* value
AT HR (bpm)	139.31 ± 17.33 (134.58-144.04)	139.75 ± 12.91 (137.92-141.58)	139.2 ± 15.74 (134.77-143.63)	138.18 ± 22.85 (131.75-144.61)	0.940
AT MET	7.41 ± 1.57 (6.98-7.84)	6.73 ± 1.47 (6.52-6.94)	5.89 ± 1.11 (5.58-6.20)	5.62 ± 1.23 (5.27-5.97)	<0.001*^,a,b,c,d,e^
ATVO_2_ (ml/kg/min)	940.5 ± 281.66 (863.62-1017.38)	1006.38 ± 343.16 (957.66-1055.10)	1060.69 ± 365.98 (957.76-1163.62)	1239.82 ± 464.2 (1109.26-1370.38)	<0.001*^,c,e^
Peak HR (bpm)	173.75 ± 17.45 (168.99-178.51)	175.96 ± 15.7 (173.73-178.19)	172.69 ± 17.75 (167.70-177.68)	173.08 ± 14.57 (168.98-177.18)	0.444
Peak MET	10.37 ± 2.22 (9.76-10.98)	9.67 ± 1.82 (9.41-9.93)	8.34 ± 1.81 (7.83-8.85)	7.81 ± 1.6 (7.36-8.26)	<0.001*^,b,c,d,e^
PeakVO_2_ (ml/kg/min)	1334.66 ± 444.52 (1213.33-1455.99)	1457.66 ± 505.72 (1385.86-1529.46)	1509.6 ± 563.7 (1351.06-1668.14)	1725.05 ± 684.68 (1532.48-1917.62)	0.002*^,c,e^
Peak VE	38.08 ± 11.1 (35.05-41.11)	41.83 ± 12.86 (40.00-43.66)	42.92 ± 14.26 (38.91-46.93)	44.4 ± 15.29 (40.10-48.70)	0.088
Peak RER	± 0.09 (1.13-1.17)	1.17 ± 0.10 (1.16-1.18)	1.17 ± 0.10 (1.14-1.20)	1.17 ± 0.10 (1.14-1.20)	0.715
PRPP	27207.89 ± 5413.23 (25730.36-28685.42)	28592.4 ± 5827.99 (27764.96-29419.84)	28748.92 ± 6294.38 (26978.60-30519.24)	30052.48 ± 6477.65 (28230.61-31874.35)	0.116
HRR	26.49 ± 9.49 (23.90-29.08)	28.83 ± 10.78 (27.30-30.36)	22.72 ± 10.11 (19.88-25.56)	24.38 ± 13.98 (20.45-28.31)	0.012*^,d^

AT MET: metabolic equivalent at anaerobic threshold; Peak MET: peak metabolic equivalent during exercise testing; AT VO_2_: oxygen consumption at anaerobic threshold; Peak VO_2_: peak oxygen consumption during exercise testing; VE: minute ventilation; RER: respiratory exchange ratio; PRPP: peak heart rate pressure product; HRR: heart rate reserve *p value < 0.05 Post-hoc Bonferroni analysis showed significant differences between the following groups: ^a^ Underweight vs. Normal, ^b^ Underweight vs. Overweight, ^c^ Underweight vs. Obesity, ^d^ Normal vs. Overweight, ^e^ Normal vs. Obesity

## Abbreviations

ACSM: American College of Sports Medicine
AT: Anaerobic Threshold
BMI: Body Mass Index
CHD: Congenital Heart Disease
CPET: Cardiopulmonary Exercise Testing
CPF: Cardiopulmonary Fitness
FMI: Fat Mass Index
HRR: Heart Rate Recovery
MET: Metabolic Equivalent
PRPP: Peak Rate Pressure Product
RER: respiratory exchange ratio
VCO2: Carbon Dioxide Output
VE/VCO₂ slope: ventilatory equivalent for CO₂
VO₂: Oxygen Consumption
VSD: Ventricular Septal Defect
